# *PLAG1* ‐Rearranged Fibromyxoid and Lipomatous Neoplasms in Children and Adults: Separate Entities or a Morphological Spectrum?

**DOI:** 10.1002/gcc.70070

**Published:** 2025-08-25

**Authors:** Vanghelita Andrei, Elena Zheleznyakova, Silvia Cavalchini, Jane Chalker, Michael Hubank, Roberto Tirabosco, Paul O'Donnell, Fernanda Amary, Adrienne M. Flanagan

**Affiliations:** ^1^ Cellular and Molecular Pathology Royal National Orthopaedic Hospital NHS Trust Middlesex UK; ^2^ Research Department of Pathology University College London Cancer Institute London UK; ^3^ Clinical Genomics The Royal Marsden Hospital NHS Trust Sutton UK; ^4^ Clinical Radiology Royal National Orthopaedic Hospital NHS Trust Middlesex UK

## Abstract

Fusions involving the *PLAG1* gene are associated with multiple cancers and benign tumors, including lipoblastoma and the more recently described pediatric fibromyxoid soft tissue tumor. We report two *PLAG1*‐rearranged mesenchymal tumors arising in adults which, although largely similar histologically to the fibromyxoid tumors reported in infants, display limited adipocytic differentiation. In both cases, the novel fusion partner was *DLEU2*. Whole genome sequencing of one of the tumors also showed loss of 13p including the *RB1* locus. Expression of PLAG1 was demonstrated by extensive immunoreactivity in both cases. We discuss the similarities of our cases to the previously described fibroblastic variants of lipoblastomas and the recently reported cases of *PLAG1*‐rearranged fibromyxoid soft tissue tumors, highlighting the overlapping morphological and molecular features. We consider that there is growing evidence that these histological entities are related to conventional lipoblastoma and represent tumors of adipocytic lineage exhibiting different stages of cellular maturation.

## 1 Introduction

*PLAG1* (pleomorphic adenoma gene 1) is located on chromosome band 8q12 and was first described in pleomorphic adenomas of the salivary glands [1]. It belongs to the PLAG family of transcription factors along with *PLAG‐like 1* and *PLAG‐like 2* genes. Rearrangement of *PLAG1* is described in lipoblastoma (LB), myoepitheliomas/mixed tumors, uterine myxoid mesenchymal tumors, and the more recently described pediatric fibromyxoid soft tissue tumor [2, 3, 4, 5, 6].

LB was first described in 1926 as a benign neoplasm of embryonal white fat, occurring predominantly in children under 3 years of age, with a wide anatomical distribution [7]. Classically, the morphology exhibits a lobular architecture with adipocytes in various stages of maturation, separated by fibrovascular septa and variable amounts of myxoid stroma [8, 9]. LBs were found to harbor *PLAG1* rearrangements in 2000 [10] and they are known to exhibit a significant variation in morphology including myxoid and lipoma‐like variants: they also present in older patients. Fritchie et al. described a series of 22 cases in patients ranging from 4 to 44 years of age, with a median of 10 years [4]. Notably, five of their cases exhibited fibromyxoid features with a minor fatty component and were labeled as “fibroblastic lipoblastomas.” Meanwhile, Chung et al. [5] identified three unusual tumors in infants (less than one of age) and genotyping led to these being classified as *PLAG1*‐rearranged fibromyxoid soft tissue tumors, reflecting their morphological appearance. Two cases lacking a fatty component were then reported in patients under 3 years of age [11, 12] and more recently, three cases with adipocytic differentiation that were also labeled as *PLAG1*‐rearranged fibromyxoid soft tissue tumors [11, 13, 14], two in adolescents and one in an adult, were published. We consider that there is now a lack of clarity as to how best to classify *PLAG1‐*rearranged tumors with and without adipocytic differentiation [5, 11, 13, 14].

Here we report two additional cases of *PLAG1*‐rearranged neoplasms with a minor adipocytic component presenting in adults that we classified as fibroblastic LB, providing evidence that these tumors are more common than hitherto recognized. The increasing number of reported *PLAG1*‐rearranged tumors exhibiting a fibromyxoid appearance with and without a fatty component, the latter in variable proportions, highlights the similarities with LBs and its variants which also can exhibit variable amounts of fibroblastic and myxoid matrix. We propose that these *PLAG1*‐rearranged tumors of soft tissue represent a spectrum of related neoplasms of adipocytic lineage at different stages of differentiation.

## 2 Case Reports

### 2.1 Case 1

A 43‐year‐old woman presented with a firm painless lump on the medial aspect of her left foot. There was no lymphadenopathy. Plain radiography showed no bony abnormality, and MRI confirmed a heterogeneous, intramuscular soft tissue mass (40 × 20 × 20 mm) abutting the plantar aspect of the first metatarsal shaft with no osseous involvement. Small foci of increased T1 signal intensity suggesting fat could be seen at the distal aspect of the mass. The resected tumor showed a well‐defined solid and firm soft tissue tumor with focal small myxoid areas.

**FIGURE 1:**
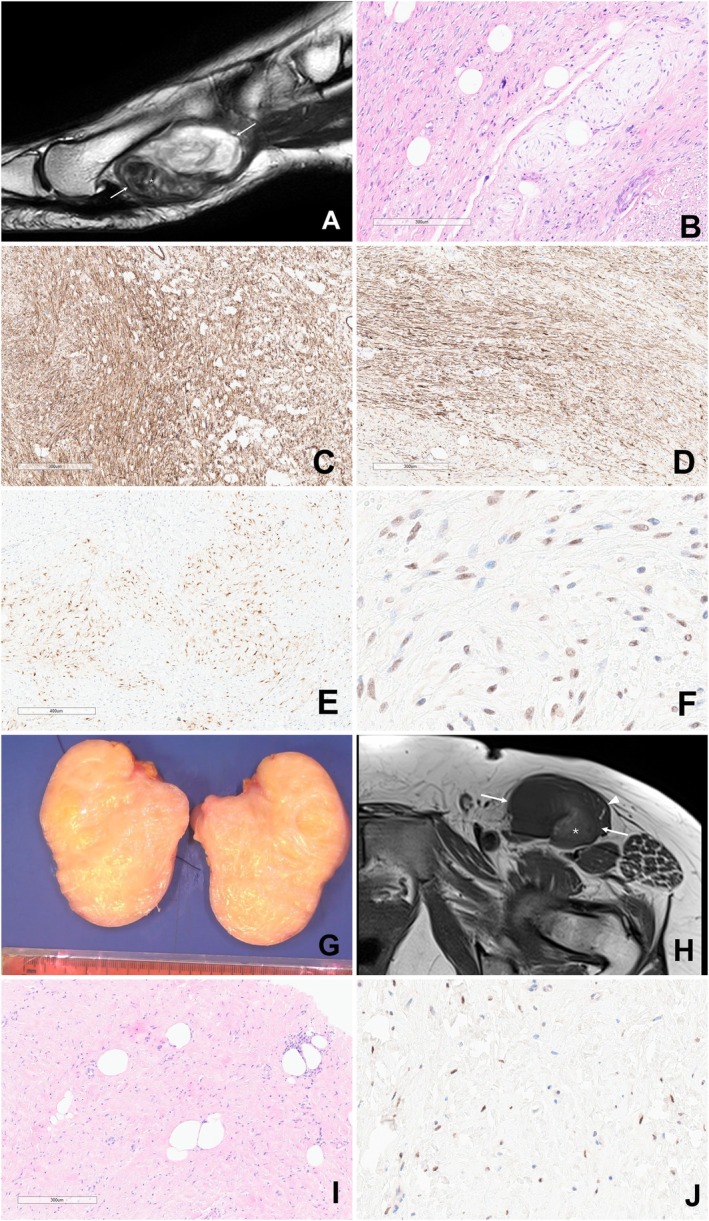
Radiology, histology, and immunohistochemical profile of the tumors. Case 1: (A) Sagittal T2‐weighted MR image of the left foot. A well‐defined mass is located in soft tissue at the plantar aspect of the first metatarsal (arrows). It is heterogeneous in signal with a hypointense component distally (*): T1 hyperintensity was also shown in this region suggesting fat. (B) Microscopy displays an overall moderately cellular tumor showing discrete myxoid nodules of low cellularity separated by areas of moderately cellular spindle cells set within a more collagenous stroma. Mature adipocytes are scattered throughout the tumor. There is scattered cytological atypia, but no mitoses or necrosis were seen. The tumor is extensively immunoreactive for (C) CD34 and (D) desmin. (E) There is focal S100 expression and (F) PLAG1 is positive in the spindle cell component. Case 2: (G) A well circumscribed multinodular lipomatous tumor with a homogeneous fatty cut surface. (H) Axial T1‐weighted fat‐saturated MR image showing a well‐defined mass in the left groin (arrows) lying deep to the sartorius muscle (arrowhead). There is subtle hyperintensity at the deep lateral aspect of the mass (*) in keeping with fat. (I) Histological examination revealed a tumor composed of bland spindle cells set in a fibrous stroma, admixed with islands of mature adipose tissue. (J) PLAG1 is positive in the spindle cell component.

The biopsy and resection specimens showed similar features revealing a tumor with moderate to high cellularity composed of fascicles of spindle cells with scattered cytological atypia, set in a fibromyxoid stroma (Figure 1). Scattered lobules of mature adipocytes were also noted in addition to a sprinkling of chronic inflammatory cells. Mitoses and tumor necrosis were not identified.

Immunohistochemistry showed the tumor cells to be extensively positive for CD34 and desmin, and scattered cells were weakly positive for S100 and EMA. Other immunomarkers (STAT6, MNF116, SMA, myogenin, MYOD1, and MUC4) were negative. H3K27me3 expression was retained.

### 2.2 Case 2

A 54‐year‐old female with a medical history of rheumatoid arthritis and Hashimoto thyroiditis presented with a painless palpable mass on the medial aspect of the left upper thigh. MRI revealed a large ovoid lesion within the posterior aspect of the left sartorius muscle, with focal extension into the inguinal canal and a fatty component in the anterolateral aspect of the lesion. On imaging, the differential diagnosis included liposarcoma because of the identification of intra‐tumoral fat and desmoid‐type fibromatosis due to low‐signal septations.

Biopsy cores and the resected specimen revealed a 95 × 75 × 58 mm paucicellular fibromyxoid tumor composed of a monomorphic population of bland spindle cells in a collagenous stroma associated with small nests of mature adipocytes (Figure 1). The spindle cell component was immunoreactive for CD34 and, to a lesser extent, for desmin. SMA, STAT6, S100, and MUC4 were negative. No *MDM2* gene amplification was detected by fluorescence in situ hybridization (FISH).

Follow‐up of both patients for 6 months showed no evidence of recurrent disease or metastasis.

## 3 Materials and Methods

The Royal National Orthopedic Hospital (RNOH) Biobank was approved by the National Research Ethics Committee of the Health Research Committee (reference: Integrated Research Application System (IRAS) project identifier: 272816). This study was approved by the National Research Ethics Committee and the UCL/UCLH Biobank Ethics Committee (project no: EC17.14).

Fluorescence in situ hybridization was performed and analyzed as previously described [15].

Formalin‐fixed paraffin‐embedded tissue samples from both cases were studied on the TruSight RNA Pan Cancer panel according to routine protocols (https://www.royalmarsden.nhs.uk/services/genomics/nhs‐healthcare‐providers). Additionally, frozen tumor and matched blood from Case 1 were subjected to whole genome sequencing (WGS) at 30× and 100× sequencing depths, respectively. These were analyzed using the cancer bioinformatics pipeline for Genome Medical Service (Cancer Genome Analysis Guide v4.0 available from FutureNHS) using GRCh38 + Decoy + HLA reference human genome. Alignment of both the tumor and germline samples was performed using the DRAGEN aligner (version 3.2.22). Germline findings were analyzed against reference panels available at NHS Genomic Medicine Service Signed OFF Panels Resource.

## 4 Results

**FIGURE 2:**
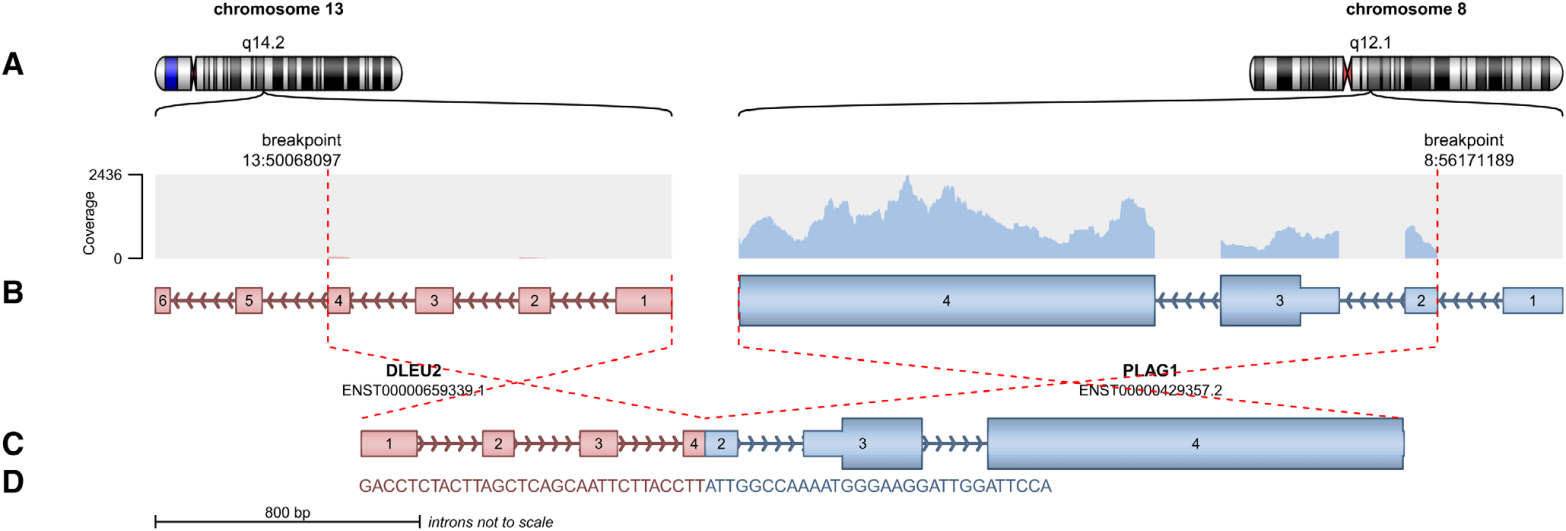
Schematic visualization of the fusion gene using Arriba software (GitHub—suhrig/arriba: Fast and accurate gene fusion detection from RNA‐Seq data). (A) Chromosome bands at the top of the graph demonstrate the breakpoint location in relation to the cytogenetic location of *DLEU2* and *PLAG1* on chromosomes 13 and 8, respectively. (B) Exonic and intronic regions of the corresponding genes, with dashed lines indicating the breakpoints and their relative genomic coordinates. (C) Visualization of the fusion gene product, containing exons 1–4 of the *DLEU2* gene and exons 2–4 of *PLAG1*. (D) The genomic sequence on each side of the breakpoint.

A *DLEU2::PLAG1* fusion with identical breakpoints was detected in both tumors by RNA‐based next‐generation sequencing (Figure 2). WGS on Case 1 detected a diploid somatic genome with segmental loss of 1p and 13p (including *RB1*) and gain of 1q. A complex rearrangement involving *CHCHD7*, *DLEU2*, *PLAG1*, and *CLSTN2* (3q23) was detected. No pathogenic germline small variants or copy number variants were identified.

## 5 Discussion

Here we report two cases of a fibromyxoid tumor in adults with a minor adipocytic component where the identification of *PLAG1* fusion, and PLAG1, CD34, and desmin immunoreactivity were supportive of the diagnosis. These findings are identical to five cases classified as “fibroblastic lipoblastomas” reported by Fritchie et al. which also presented in children over 4 years of age and in adults. By incorporating genetic testing with histological features, this publication highlighted the range of features observed in *PLAG1*‐rearranged LB. They grouped the LB on the basis of their histological appearance as “conventional” with “characteristic lobules of adipocytes in various stages of maturation in a myxoid stroma with a fine capillary vascular network”; “maturing” with “variably sized lobules of mature adipose tissue partitioned by thin fibrous septa without myxoid stroma”; and “fibroblastic” with “bland spindled to plump ovoid cells embedded in a fibrous stroma with a vaguely plexiform arrangement of small myxoid and adipocytic nodules.” The study demonstrated that they occur over a wider age range than previously accepted. Since then, LB with canonical *PLAG1* gene rearrangement in adults have been reported by others [16, 17].

*PLAG1*‐rearranged fibromyxoid soft tissue tumors without adipocytic differentiation were described in an assiduous study of three tumors presenting in infants with a distinctive uniform histological appearance [5]. Although the classical variant of LB is recognized as having a diverse range of histological features in different proportions, including a fibromyxoid matrix and a conspicuous immature spindle cell component, the complete absence of fat had not been reported. Nevertheless, Chung et al. recognized similarities with the fibroblastic LB in adults described by Fritchie et al. and considered that they may be related tumors. Since then, *PLAG1*‐rearranged fibromyxoid soft tissue tumors have been reported in older patients [11, 14, 17].

The papers by Fritchie et al. and Chung et al. have stimulated recent interest in LB and have resulted in a greater awareness and knowledge of these rare tumors. Based on the increasing number of reported cases sharing *PLAG1* rearrangements, evidence is growing that the histological features are more varied and overlapping more commonly than previously recognized [9, 16, 17, 18]. Taken together, the evolving picture of these tumors suggests that they represent tumors of adipocytic lineage exhibiting features of different stages of cellular differentiation.

Although the number of cases remains small and long‐term follow‐up is limited, there is no evidence to date that any of the histological variants behave differently. They have been reported to exceed 100 mm, but the literature indicates that they are slow‐growing, a feature that was highlighted in a report of a mass in a 13‐year‐old patient that had been present for over 10 years [13]. The tumor also displayed indolent behavior in both of our cases. Although recurrence is not uncommon [18, 19, 20], there are no reports of malignant transformation. The site of presentation may go some way to explain the incidence of relapse: many occur at sites from where it is difficult to achieve complete resection, such as the head and neck, mediastinum, and retroperitoneum [18, 19, 20].

WGS analysis was available in one of our *PLAG1*‐rearranged cases and revealed loss of *RB1*, a finding previously unreported in either LB or fibromyxoid soft tissue tumors. However, loss of RB1 expression and deletion of 13q14 is a unifying common event in several other tumors in which fatty differentiation and CD34 expression occur and therefore should be considered in the differential diagnosis of *PLAG1*‐rearranged LB (conventional, maturing, and fibroblastic variants), including lipomas that are almost completely composed of mature fat, spindle cell/pleomorphic lipomas, atypical spindle cell/pleomorphic lipomatous tumor, mammary‐type myofibroblastoma, and cellular angiofibroma [21, 22, 23, 24]. Other tumors that can also be mistaken for LB include myxoid liposarcoma, atypical lipomatous tumor/well differentiated liposarcoma, and low‐grade fibromyxoid sarcoma, which can be distinguished by failing to detect their recurrent alterations including *DDIT3* fusions, *MDM2/CDK4* amplification, and *FUS::CREB3L2/1* fusions, respectively.

In our two cases, *PLAG1* was fused with a novel partner, *DLEU*2, adding to the growing number of reported PLAG1 fusion gene partners, which include *SRSF3*, *PCMTD1*, *YWHAZ*, *CTDSP2*, *PPP2R2A*, *COL1A2*, *COL3A1*, *HAS2*, *RAD51L1*, *RAB2A*, *BOC*, *CHCHD7*, *SRSF3*, *HNRNPC*, *PCMTD1*, *EEF1A1*, *YWHAZ*, *CTDSP2*, *PP2R2A*, *DDX6*, *KLF10*, *KANSL1L*, *PI15* (8q21.13), and *ZEB* (2q22.3). The underlying genetic changes involving all fusion transcripts are remarkably similar and involve promoter swapping, resulting in expression of PLAG1 [25].

Antibodies against PLAG1 are now commercially available and detect PLAG1 expression in *PLAG1*‐rearranged tumors, facilitating the diagnosis of LB and other tumors expressing this fusion. However, the expression can be focal and therefore the absence, particularly in a biopsy, is not informative. Desmin immunoreactivity can also be helpful in differentiating LB from its mimics, but it can be focal, diffuse, or absent.

The studies by Fritchie and Chung represent another example of how correlation of even small numbers of tumors with similar morphological phenotypes with their genotypes has improved diagnostic accuracy [5]. Recognizing the morphological features remains the cornerstone of tissue diagnosis, particularly if access to sophisticated molecular testing is limited.

## 6 Conclusion

Here we have reported two additional *PLAG1*‐rearranged fibromyxoid soft tissue tumors with the same novel fusion partner, presenting in adults, in which mature adipocytic differentiation represents a minor component. We consider that they are likely to represent part of a spectrum of histological features seen in LB (ranging from conventional to fibroblastic variants), fibromyxoid tumors of soft tissue in children less than 3 years old and sometimes older, and maturing LBs.

## Author Contributions

A.M.F. designed the study. E.Z., S.C., and J.C. performed molecular analysis. A.M.F., and M.H. wrote the manuscript with support from V.A. All authors discussed and contributed to the final manuscript.

## Ethics Statement

The Royal National Orthopedic Hospital (RNOH) Biobank was approved by the National Research Ethics Committee of the Health Research Committee (reference: Integrated Research Application System (IRAS) project identifier: 272816). This study was approved by the National Research Ethics Committee and approved by the UCL/UCLH Biobank Ethics Committee (project no: EC17.14).

## Conflicts of Interest

The authors declare no conflicts of interest.

## Data Availability

The data that support the findings of this study are available from the corresponding author upon reasonable request.
